# Assessing the temporal within-day glycemic variability during hospitalization in patients with type 2 diabetes patients using continuous glucose monitoring: a retrospective observational study

**DOI:** 10.1186/s13098-024-01269-0

**Published:** 2024-03-01

**Authors:** Ying Xing, Min Wu, Hongping Liu, Penghui Li, Guoming Pang, Hui Zhao, Tiancai Wen

**Affiliations:** 1 Institute of Information on Traditional Chinese Medicine, China Academy of Chinese Medical Sciences, Beijing, China; 2 Traditional Chinese Medicine Data Center, China Academy of Chinese Medical Sciences, Beijing, China; 3 Kaifeng Traditional Chinese Medicine Hospital, Henan, China; 4 China Center for Evidence-Based Traditional Chinese Medicine, China Academy of Chinese Medical Sciences, Beijing, China

**Keywords:** Within-day glycemic variability, Glycemic fluctuations, Glycemic stability, Type 2 diabetes

## Abstract

**Aims:**

Frequent and extensive within-day glycemic variability (GV) in blood glucose levels may increase the risk of hypoglycemia and long-term mortality in hospitalized patients with diabetes. We aimed to assess the amplitude and frequency of within-day GV in inpatients with type 2 diabetes and to explore the factors influencing within-day GV.

**Methods:**

We conducted a single-center, retrospective observational study by analyzing hospital records and 10-day real-time continuous glucose monitoring data. Within-day GV was assessed using the coefficient of variation (%CV). The primary outcome was the amplitude and frequency of within-day GV. The frequency of within-day GV was assessed by the consecutive days (CD) of maintaining within the target %CV range after first reaching it (CD after first reaching the target) and the maximum consecutive days of maintaining within the target %CV range (Max-CD). The target %CV range was less than 24.4%. We evaluated the factors influencing within-day GV using COX regression and Poisson regression models.

**Results:**

A total of 1050 cases were analyzed, of whom 86.57% reduced the amplitude of within-day GV before the sixth day of hospitalization. Of the 1050 hospitalized patients, 66.57% stayed within the target %CV range for less than two days after first reaching the target and 69.71% experienced a Max-CD of fewer than four days. Reducing the average postprandial glucose excursion (hazard ratio [HR]: 0.81, 95% confidence interval [CI]: 0.77–0.85; incidence rate ratios [IRR]: 0.72, 95% CI: 0.69–0.74) and the use of α-glucosidase inhibitors (IRR: 1.1, 95% CI: 1.01–1.18) and glucagon-like peptide-1 agonist (IRR: 1.30, 95% CI: 1.02–1.65) contributed to reducing the amplitude and decreasing the frequency of within-day GV. However, the use of insulin (HR: 0.64, 95% CI: 0.55–0.75; IRR: 0.86, 95% CI: 0.79–0.93) and glinide (HR: 0.47, 95% CI: 0.31–0.73; IRR: 0.84, 95% CI: 0.73–0.97) may lead to an increased frequency of within-day GV.

**Conclusions:**

An increasing frequency of within-day GV was observed during the hospitalization in patients with type 2 diabetes, despite the effective reduction in the amplitude of within-day GV. Using medications designed to lower postprandial blood glucose could contribute to minimize the risk of frequent within-day GV.

**Supplementary Information:**

The online version contains supplementary material available at 10.1186/s13098-024-01269-0.

## Introduction

For a long time, glycated hemoglobin (HbA1c) has been viewed as a standard marker reflecting the level of glycemic control in patients with type 2 diabetes (T2D) [1, 2]. Achieving HbA1c values of 7% or less is associated with a decreased risk of micro- and macrovascular complications [3, 4]. However, by definition, HbA1c, reflects average blood glucose levels over the preceding 2–3 months, does not account for daily acute fluctuations. Those fluctuations in blood glucose are termed glycemic variability (GV). Thus, patients with T2D can still experience significant GV even when reaching target HbA1c levels [5, 6].

GV measures the extent of fluctuation in glucose levels over time. It primarily encompasses two components: amplitude, indicative of the magnitude of blood glucose excursions, and frequency (timing), denoting the time intervals during which these fluctuations occur [7]. Within-day GV refers to glucose fluctuations from peaks to nadirs within a single day [8]. Peaks, especially in T2D, usually correspond to postprandial hyperglycemia, while nadirs can indicate the risk of hypoglycemia [5]. Both the amplitude and the frequency of GV contribute to the risks for hypoglycemia and postprandial hyperglycemia associated with diabetes [9].

In a hospitalized setting, strict glycemic management ensures that uncontrolled T2D patients are less vulnerable to hyperglycemia’s detrimental effects [10]. However, swift reductions in overall blood glucose levels may increase within-day GV and thus potentiate the risk of hypoglycemia [11, 12]. Given this, it is imperative to monitor within-day GV, including amplitude and frequency, in hospitalized T2D patients. Accumulating evidence underscores the pivotal role of curtailing within-day GV amplitude in minimizing hypoglycemic risk [13–16]. However, present-day research is somewhat remiss in shedding light on the frequency of GV [12].[17]. Studies indicated that acute glycemic fluctuations within a day induce more oxidative stress compared to chronic hyperglycemia, believed to be the primary underlying mechanism behind glucose-induced vascular damage. This insight lends credence to exploring the hypothesis that recurrent acute glycemic fluctuations over time might amplify oxidative stress. Namely for healthcare practitioners, a continuous assessment of within-day GV stability could be more consequential than solely mitigating chronic hyperglycemia. Additionally, several studies have shown that increased GV is independently associated with extended hospitalizations, augmented short-term and long-term mortality in diabetic and critically ill or non-critically ill hospitalized patients [18–22]. Therefore, for hospitalized T2D patients, a dual evaluation approach focusing on both the amplitude of within-day GV and its temporal stability is crucial. The intent is to alleviate the accumulative strain of repetitive acute glycemic peaks and troughs. By dynamically adapting treatment modalities in sync with daily GV patterns, the overarching goal is to harmonize reduced hyperglycemia, limited hypoglycemia, and stable GV, culminating in ideal blood glucose control. However, current literature seems to be scant in its coverage of our knowledge. There is currently insufficient available evidence regarding the temporal within-day glycemic variability among hospitalized T2D patients with type 2 diabetes.

Using 10-day real-time continuous glucose monitoring (rtCGM) data, we scrutinized the amplitude variations of Within-day GV in 1050 hospitalized patients, and observed the frequency of Within-day GV based on several additional outcome measures. Furthermore, we conducted a regression model analysis to examine the influencing factors on both the amplitude and frequency of Within-day GV.

## Methods

### Data sources and study design

This retrospective, single-center observational study was conducted at the Kaifeng Hospital of Traditional Chinese Medicine in Henan Province, China. We performed a retrospective analysis of the hospital records and 10-day rtCGM (iPro(®)2 CGM system, Medtronic MiniMed, Northridge, CA) data for patients with T2D who were admitted due to poor glycemic control between January 1, 2018, and June 16, 2021. Only adult patients (aged ≥ 18 years) with type 2 diabetes who underwent rtCGM for more than 10 days were eligible for inclusion in the study. To investigate the factors influencing the temporal stability of within-day GV, we considered variables such as age, gender, duration, body mass index (BMI), low density lipoprotein (LDL), HbA1c, C-peptide, comorbidities, treatment modalities (oral antidiabetic drugs and insulin), and specific rtCGM data metrics (24-hour mean glucose, standard deviation of glucose, fasting plasma glucose (FPG), and postprandial glucose excursion (PPGE)). The baseline for the study was defined as 24 h after patient admission. All laboratory tests were drawn in a fasting state from the blood samples within 24 h after admission. All treatments were performed by the experienced physician after blood glucose profile assessments.

### GV measurements and outcome assessments

Coefficient of variation (CV) was considered the most appropriate measure for evaluating GV [9]. CV was calculated as %. In this study, within-day %CV (%CV_w_) was used to evaluated within-day GV, with the within-day standard deviation (SD_w_) of glucose (mean SD of all measurements within a 24-h period) divided by the 24-hour mean glucose (Mean) [23]. The formula for calculating %CV_w_ is shown in Eq. (1).

1 $$ {\text{\% }}C{V_w} = \frac{{S{D_w}}}{{Mean}} \times 100$$

**Fig. 1:**
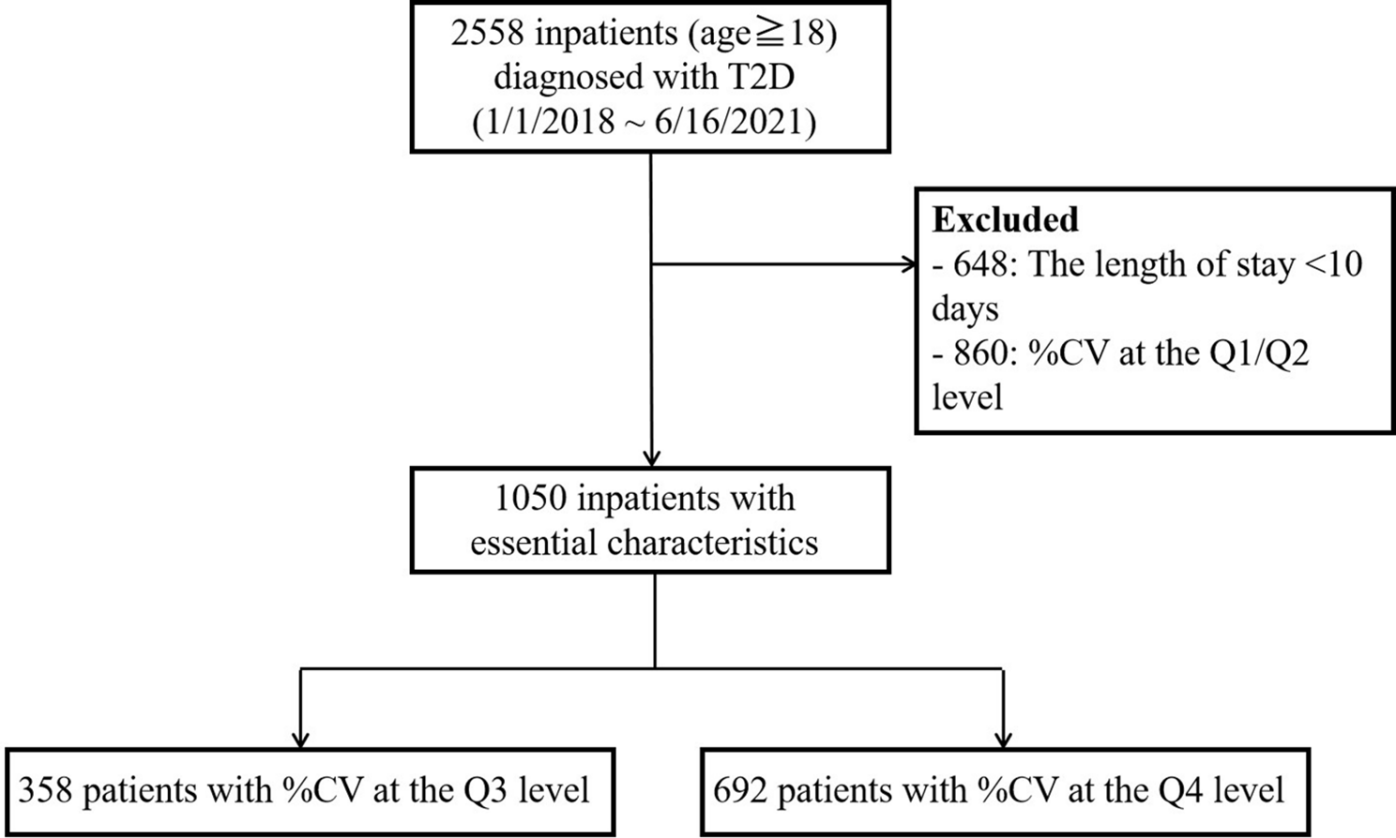
Flowchart of study population selection

According to previous research [24], a threshold of 33% for %CV was established as an indicator of excessive GV in Chinese patients with diabetes. However, this value was primarily used to differentiate the upper limit of %CV between stable and unstable GV. Rodbard et al. [25] proposed the utilization of regions defined by the 25th, 75th, and 75th percentiles of %CV to categorize GV levels as excellent, good, fair, and poor. This approach allowed for a more precise evaluation of patients’ GV compared to a “one-size-fits-all”, such as a 33% threshold. In line with the findings of Mo et al. [24], %CV in Chinese patients with T2D was divided into four groups based on quartiles: excellent (≤ 19.3%), good (19.4–24.4%), fair (24.5–30.4%), and poor (> 30.4%). Our study included patients with baseline %CV rated as “fair” (Cohort 1) and “poor” (Cohort 2). Our final study population included 1050 patients (Fig. 1). The quartile levels of “excellent” and “good” for %CV, which were our study’s target %CV range, have been deemed more favorable for reducing the risk of hypoglycemia in T2D patients [26].

The primary outcome was the amplitude and frequency of within-day GV in hospitalized patients with type 2 diabetes. The design ideas of this research, according to Braithwaite’s proposed framework [12], were shown as follows: (1) the amplitude of within-day GV: aimed at investigating the likelihood and cumulative incidence of reaching the target %CV range for the first time, (2) frequency of within-day GV: aimed at assessing the continuous days of maintaining within the target %CV range, including the consecutive days (CD) of maintaining within the target %CV range after first reaching the target (CD after first reaching the target) and the maximum consecutive days of maintaining within the target %CV range (Max-CD) during the 10-day period. Furthermore, We considered the SD of %CV values and the proportion of Max-CD as additional measures of timing of within-day GV. The proportion of MAX-CD was defined as MAX-CD divided by the the remaining hospital days. The remaining hospital days were calculated as the total number of observational days (10 days) minus the number of days required to reach the target %CV range for the first time. The secondary objective was to determine the factors associated with amplitude and frequency of within-day GV. Additionally, we assessed the overall glycemic control via FPG and PPGE, while the PPGE provided insights into postprandial glycemic dynamics [27].

The continuous variables like diabetes duration (0 to ≤ 3, 3 to ≤ 10, and >10 years), LDL (< 2.6, and ≥ 2.6 mmol/L), and C-peptide (< 2.5, and ≥ 2.5 ng/mL) obtained from the baseline period were categorized into categorical variable. According to the consensus of Chinese experts on medical nutrition therapy for overweight/obesity [28], Participants were classified as normal weight (18.5 ≤ BMI ≤ 23.9 kg/m2), overweight (24 to ≤ BMI ≤ 27.9 kg/m2) and obesity (BMI ≥ 28 kg/m2). The age and HbA1c at baseline were calculated as continuous variables. The average FPG and PPGE during the 10-day period were also computed as continuous variables. To evaluate the influence of comorbidities on GV, 9 prevalent comorbidities in diabetes were included, such as liver diseases, hypertension, hyperlipidemia, coronary artery atherosclerosis, chronic kidney disease, cerebral infarction, ischemic cerebrovascular disease, diabetic retinopathy, diabetic polyneuropathy. Each comorbidity was treated as a categorical variable.

Antidiabetic medications administered during hospitalization were classified as follows: dipeptidyl peptidase-4 inhibitors (DPP-4i), sulfonylurea, metformin, α-glucosidase inhibitors, SGLT2 inhibitors (SGLT2i), thiazolidinediones (TZDs), glinide, glucagon-like peptide-1 (GLP-1) agonist, and insulin. In our research, each antidiabetic medication was considered a categorical variable.

### Statistical analysis

Descriptive analysis was used to evaluate admission characteristics. Continuous variables were reported as mean ± SD if normally distributed, alternatively as median with interquartile range (IQR). Categorical variables were reported as frequencies and percentages.

We employed the Kaplan-Meier method to delineate the cumulative incidence rate of both patient cohorts, achieving the target %CV range for the first time. Differences between the two cohorts were assessed using the log-rank test, with a significance level set at *p* < 0.05. We used descriptive statistics to calculate the CD after first reaching the target, as well as the MAX-CD and the proportions of MAX-CD for each patient. The SD of %CV values during hospitalization for each patient was calculated and presented graphically.

Cox proportional hazards regression model was used to determine the risk factors affecting the number of days required to reach the target %CV range for the first time. As for factors that might affect timing of within-day GV, logistic and Poisson regression models were used for evaluation. The primary analysis involves using a Poisson regression model to determine the influencing factors on the MAX-CD. In the sensitivity analysis, we employed a logistic regression model to analyze the factors influencing the proportions of MAX-CD and the SD of %CV values as additional outcomes. The results were visualized in forest plots, showing the hazard ratio (HR, for Cox regression), incidence rate ratios (IRR, for Poisson regression) and odds ratios (OR, for logistic regression) alongside the 95% confidence interval (CI) for each outcome. All analyses were carried out with R 4.3.1.

## Results

The study cohort consisted of 1050 individuals: 358 patients in Cohort 1 and 692 in Cohort 2. Participant demographics and baseline characteristics were similar across the two study groups. Most participants were male (62.3% for Cohort 1 and 57.9% for Cohort 2), overweight or obese (74.0% for Cohort 1 and 68.2% for Cohort 2), with a disease duration of fewer than 3 years or 3–10 years (75.7% for Cohort 1 and 73% for Cohort 2). Subjects in Cohort 1 (56.3 ± 14.0 years) had a slightly younger average age than those in Cohort 2 (59.5 ± 13.1 years), while higher median HbA1c values were reported in Cohort 1 (8.5% [69.4 mmol/mol]). (Table 1).

The most prevalent comorbidities were diabetic polyneuropathy (48.3%), hypertension (42.7%), and chronic kidney disease (32.6%). The most frequently used antidiabetic medications were insulin (58.1%), α-glucosidase inhibitors (31.4%), and TZDs (21.1%) (Table 1).

**Table 1:** Participant characteristics

Characteristics	Cohort 1(*N* = 358)	Cohort 2(*N* = 692)
**Sex**		
Female	135 (37.7%)	291 (42.1%)
Male	223 (62.3%)	401 (57.9%)
**Age (years, mean ± SD)**	56.3 ± 14.0	59.5 ± 13.1
**HbA1c** ^*^ **(%, median (IQR))**	8.5 [69.4 mmol/mol] (7.6 [59.9 mmol/mol], 10.3 [89.1 mmol/mol])	8.3 [67.2 mmol/mol] (7.1 [54.1 mmol/mol], 10.0 [85.8 mmol/mol])
**BMI** ^**£**^ **(kg/m** ^**2**^ **)**		
Normal weight	93 (26%)	220 (31.8%)
Overweight	194 (54.2%)	378 (54.6%)
Obesity	71 (19.8%)	94 (13.6%)
**Diabetes duration (year)**		
0 to ≤ 3	140 (39.1%)	263 (38.0%)
3 to ≤ 10	131 (36.6%)	249 (36.0%)
>10	87 (24.3%)	180 (26.0%)
**C-peptide (ng/mL)**		
<2.5	146 (40.8%)	315 (45.5%)
≥ 2.5	212 (59.2%)	377 (54.5%)
**LDL** ^**§**^ **(mmol/L)**		
<2.6	108 (30.2%)	272 (39.3%)
≥ 2.6	250 (69.8%)	420 (60.7%)
**Liver diseases**	71 (19.8%)	108 (15.6%)
**Hypertension**	145 (40.5%)	303 (43.8%)
**Hyperlipidemia**	52 (14.5%)	85 (12.3%)
**Coronary artery atherosclerosis**	78 (21.8%)	154 (22.3%)
**Chronic kidney disease**	112 (31.3%)	230 (33.2%)
**Cerebral infarction**	37 (10.3%)	117 (16.9%)
**Ischemic cerebrovascular disease**	39 (10.9%)	77 (11.1%)
**Diabetic retinopathy**	60 (16.8%)	135 (19.5%)
**Diabetic polyneuropathy**	164 (45.8%)	343 (49.6%)
**DPP-4i** ^**¥**^	46 (12.9%)	64 (9.3%)
**Glinide**	26 (7.3%)	69 (10.0%)
**GLP-1 agonist** ^**¢**^	6 (1.7%)	5 (1.0%)
**Insulin**	190 (53.1%)	420 (60.7%)
**Metformin**	20 (5.6%)	43 (6.2%)
**SGLT2i** ^**#**^	33 (9.2%)	42 (6.1%)
**Sulfonylurea**	40 (11.2%)	84 (12.1%)
**TZDs** ^**¶**^	76 (21.2%)	146 (21.1%)
**α-glucosidase inhibitors**	101 (28.2%)	229 (33.1%)

£BMI: body mass index

*HbA1c: glycated hemoglobin

§LDL: low density lipoprotein

¥DPP-4i: dipeptidyl peptidase-4 inhibitors

#SGLT2i: SGLT2 inhibitors

¶TZDs: thiazolidinediones

¢GLP-1 agonist: glucagon-like peptide-1 agonist

### Overall glycemic control

**Fig. 2:**
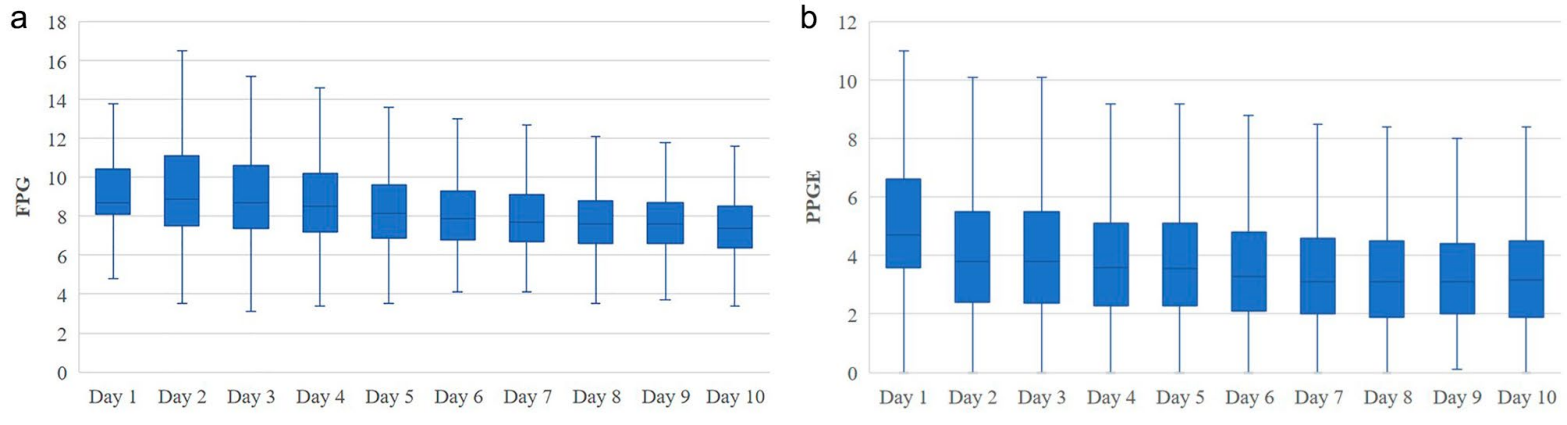
Within-day FPG and PPGE values differ over time after hospital admission. The x-axis represents the hospitalization day and the y-axis denotes the FPG or PPGE values. Boxplots were drawn at each hospitalization day using the FPG or PPGE values of all patients

From day 1 to day 10, median (IQR) FPG decreased from 8.7 mmol/L (8.1 mmol/L, 10.4 mmol/L) to 7.4 mmol/L (6.4 mmol/L, 8.5 mmol/L) (*p* < 0.01), and median (IQR) PPGE decreased from 4.7 mmol/L (3.6 mmol/L, 6.6 mmol/L) to 3.15 mmol/L (1.9 mmol/L, 4.5mmol/L) (*p* < 0.01) in all paticipants. (Fig. 2)

### Amplitude of within-day GV: the cumulative incidence of first reaching the target %CV range

**Fig. 3:**
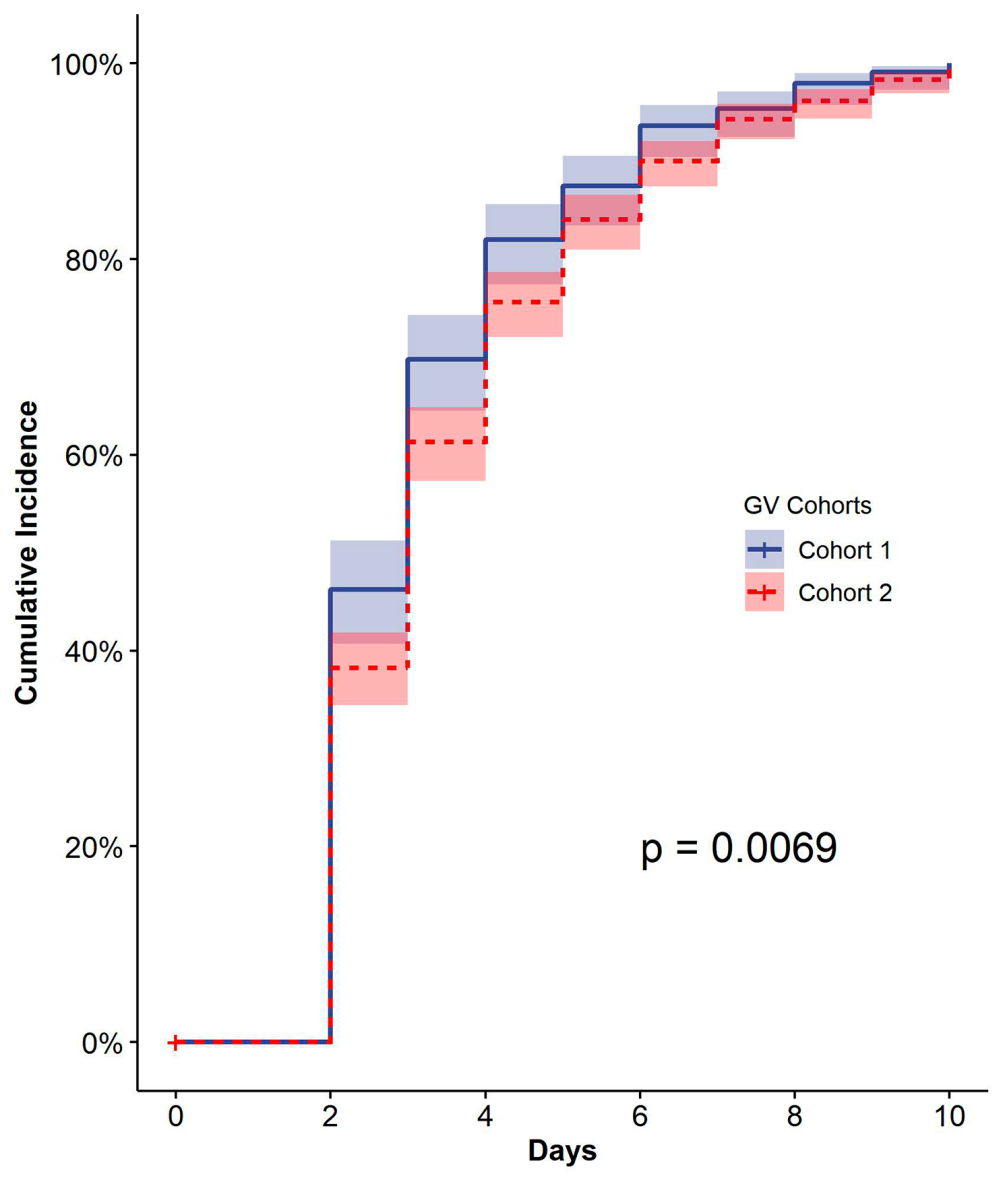
Cumulative incidence of reaching the target %CV range for the first time. The x-axis represents the hospitalization day and the y-axis represents the cumulative incidence values

Over the 10-day observation period, the estimated probability of reaching the target %CV range for the first time was highest on Day 2 (38.9%; Table 2). Nearly all (94.8%) of the patients reached the target %CV range for the first time within 10 days (Table 2). From day 1 to day 6, 86.5% of the patients reached the target %CV range for the first time. The cumulative probability of first reaching the target %CV range remained stable from day 6 to day 10 (Table 2). The cumulative incidence of first reaching the target %CV range was higher in cohort 1 compared to cohort 2 (*P* <0.01; Fig. 3).

**Table 2:** Probability of reaching the target %CV range for the first time

Days of admission, day	Participants who had not reached the target %CV range, n	Participants who first reached the target %CV range, n	Estimated probability	Cumulative probability
1	1050	0	0	0
2	642	408	38.9%	38.9%
3	411	231	22%	60.9%
4	276	135	12.9%	73.7%
5	202	74	7.0%	80.8%
6	142	60	5.7%	86.5%
7	108	34	3.2%	89.7%
8	87	21	2.0%	91.7%
9	69	18	1.7%	93.4%
10	55	14	1.3%	94.8%

### Frequency of within-day GV: the continuous days of maintaining within the target %CV range

Of the 1050 hospitalized patients, 66.6% stayed within the target %CV range for less than two days after first reaching the target and 69.7% experienced a Max-CD of fewer than four days. (Table 3 and Supplementary Material Table S1)

The average remaining hospitalization days for 1050 patients after first reaching the target %CV range was 7 days, whereas 63.9% of patients had MAX-CD less than or or equal to half of the remaining hospitalization days (The proportion of MAX-CD less than or equal to 50% was observed in 63.9% of the patients.). (Table 3 and Supplementary Material Table S1)

**Fig. 4:**
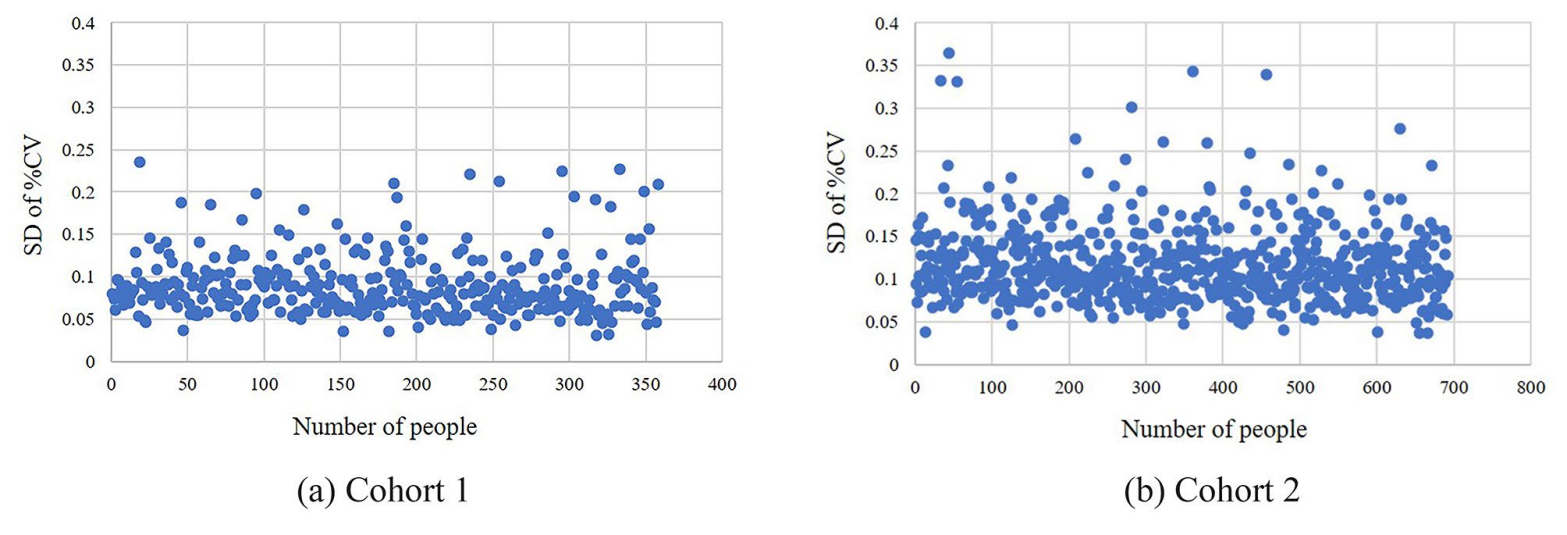
The SD of the %CV values for patients in two cohorts. The x-axis represents the number of people and the y-axis represents the SD of the %CV values

67.3% of patients in Cohort 1 had a SD of %CV ranging between 0.05 and 0.1 (Fig. 4a), while 82.1% of patients in Cohort 2 exhibited a SD of %CV within the range of 0.05 to 0.15 (Fig. 4b).

**Table 3:** Analysis of frequency of within-day GV

Analysis	Cohort 1	Cohort 2	Total
CD^*^ after first reaching target^&^			
0–2 days	212 (59.2%)	487 (70.4%)	699 (66.6%)
≥ 3 days	146 (40.8%)	205 (29.6%)	351 (33.4%)
Max-CD^$^			
0–4 days	228 (63.7%)	504 (72.8%)	732 (69.7%)
≥ 5 days	130 (36.3%)	188 (27.2%)	318 (30.3%)
The proportion of MAX-CD^#^			
≤ 50%	206 (57.6%)	465 (67.2%)	671 (63.9%)
>50%	152 (42.5%)	227 (32.8%)	379 (36.1%)

*CD: consecutive days

^&^CD after first reaching target: the consecutive days of maintaining within the target %CV range after first reaching target

^$^Max-CD: the maximum consecutive days of maintaining within the target %CV range

^#^The proportion of MAX-CD: was defined as MAX-CD divided by the the remaining hospital days. The remaining hospital days were calculated as the total number of observational days (10 days) minus the number of days required to reach the target %CV range for the first time.

#### Factors influencing the within-day GV

**Fig. 5:**
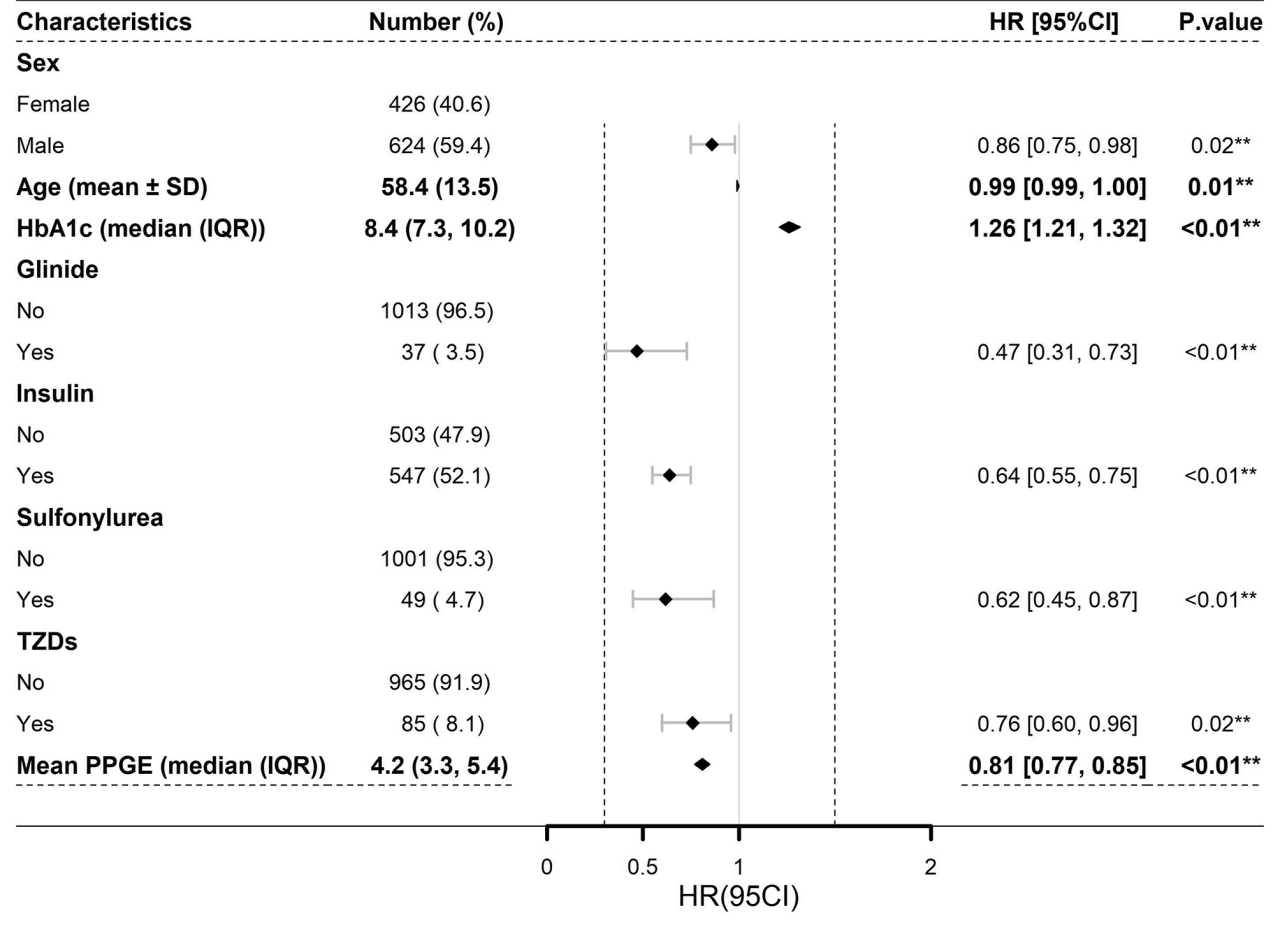
Factors influencing the number of days required to reach the target %CV range. **P < 0.05. CI, confidence interval; HR, hazard ritio

According to the COX proportional hazards model, factors influencing the number of days required to reach the target %CV range encompass sex, age, HbA1c, use of glinide, insulin, sulfonylurea and TZDs, and the mean PPGE. Male patients (HR: 0.86, 95% CI: 0.75–0.98, *P* < 0.05) were less likely to achieve the target %CV range for the first time than female patients. The likelihood of reaching the target %CV range for the first time diminished with advancing age (HR: 0.99, 95% CI: 0.99-1.00, *P* < 0.05). Individuals with higher levels of HbA1c had a higher probability of reaching the target %CV range for the first time (HR: 1.26, 95% CI: 1.21–1.32, *P* < 0.05). Conversely, the usage of Glinide (HR: 0.47, 95% CI: 0.31–0.73, *P* < 0.05), Insulin (HR: 0.64, 95% CI: 0.55–0.75, *P* < 0.05), Sulfonylurea (HR: 0.62, 95% CI: 0.45–0.87, *P* < 0.05) and TZDs (HR: 0.76, 95% CI: 0.60–0.96, *P* < 0.05) markedly reduced this probability. Moreover, an increasing mean PPGE value reduced the likelihood of reaching the target %CV range for the first time (HR: 0.81, 95% CI: 0.77–0.85, *P* < 0.05)(Fig. 5).

**Fig. 6:**
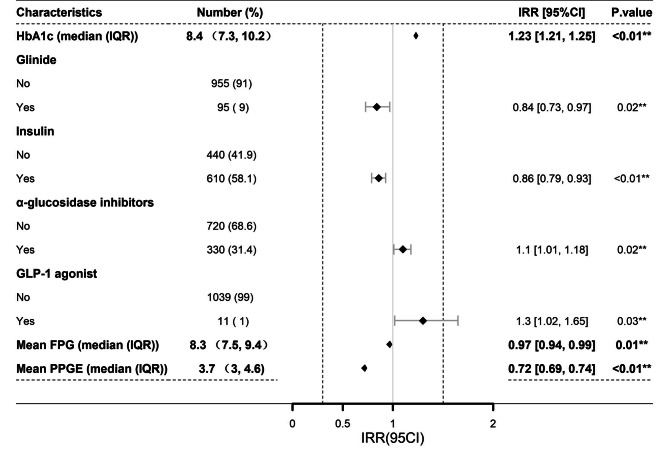
Factors influencing the MAX-CD. **P < 0.05. CI, confidence interval; IRR, incidence rate ratios

From the Poisson regression analysis, the principal factors affecting the maximum consecutive days of staying within the target %CV range (Max-CD) were HbA1c, use of glinide, insulin,α-glucosidase inhibitors and GLP-1 agonists, along with the mean FPG and PPGE. Patients with higher HbA1c values were more likely to have an increased MAX-CD (IRR: 1.23, 95% CI: 1.21–1.25, *P* < 0.05). Similarly, the use of α-glucosidase inhibitors (IRR: 1.10, 95% CI: 1.01–1.18, *P* < 0.05) and GLP-1 agonists (IRR: 1.30, 95% CI: 1.02–1.65, *P* < 0.05) contributed to increasing the MAX-CD. Conversely, the administration of glinide (IRR: 0.84, 95% CI: 0.73–0.97, *P* < 0.05) and insulin (IRR: 0.86, 95% CI: 0.79–0.93, *P* < 0.05), along with an increase in average FPG (IRR: 0.97, 95% CI: 0.94–0.99, *P* < 0.05) and PPGE (IRR: 0.72, 95% CI: 0.69–0.74, *P* < 0.05) values, may contribute to a decrease in the MAX-CD (Fig. 6). Sensitivity analysis showed similar results (Supplementary Figs. S1 and S2).

## Discussion

We observed that glycemic control of all hospitalized patients with type 2 diabetes, assessed by FPG and PPGE, was effectively managed. An impressive 86.5% of patients successfully managed to lower their within-day GV amplitude by the sixth day of hospitalization, however, a concomitant increase in the frequency of within-day GV is observed. Upon admission, patients with higher within-day GV typically experienced relatively shorter durations of stable within-day GV.

Medical institutions prioritize reducing FPG and PPGE to address hyperglycemia, given their significant contribution to overall hyperglycemia [29, 30]. Increasingly, evidence suggests that hyperglycemia activates oxidative stress (OS) by hyperglycemia through various pathways, significantly contributing to micro- and macrovascular complications in T2D [31–34]. However, past research has indicated that glucose fluctuations, including within-day GV, might exert more adverse effects than persistent hyperglycemia on OS [17, 35, 36], a crucial factor in exacerbating diabetic cardiovascular complications. Several studies also suggested that short-term GV could heighten the risk of adverse clinical outcomes in individuals with T2D [37–40]. Another study highlighted that short-term GV holds more weight than the mean daily glucose concentration in predicting the risk of unequivocal hypoglycemia, a pressing issue in enhancing the life quality of T2D patients [41]. Such insights underscore the need for patients with T2D, that is, persons with diabetes should strike a balance between managing chronic hyperglycemia and curbing glucose fluctuations. This would minimize both the long-term risk of developing diabetic complications and the acute hypoglycemia risks [42]. However, our results indicate that achieving this balance remains difficult.

The key to breaking this balance was the volatile shift of within-day GV from peaks to troughs. We found a robust correlation between within-day GV stability and factors like use of antidiabetes drugs, PPGE, and HbA1c values. Our data indicates that heightened postprandial glucose spikes were linked to increased within-day GV. Echoing the findings of Monnier et al. [43] and Candido et al. [44], half of the overall within-day GV resulted from postprandial glucose spikes. It is plausible to conclude that PPGE is pivotal in the intricate dynamics between within-day GV and factors like HbA1c. Our data suggests that patients with higher HbA1c values are conducive to stabilizing within-day GV. This is corroborated by findings from an observational study indicating that the contribution of PPGE to total glucose decreases as HbA1c increases [45]. Several studies have arrived at similar conclusions [30, 46–48].

Furthermore, regarding the interplay between within-day GV and therapeutic drugs in T2DM medications, our findings align with previous studies underscoring the efficacy of GLP-1 agonists in curtailing postprandial glucose spikes and overall within-day GV [49, 50]. However, we also discerned a positive link between high within-day GV fluctuations and the use of sulfonylurea, insulin, and glinide due to their heightened hypoglycemia risks [51]. This association is consistent with extensive literature suggesting that hypoglycemia risks are pronounced with insulin therapy, both basal insulin and intensive strategies [52–55], sulfonylurea [56–58], and glinide [59–61].

Our observations were partially congruent with findings from two studies [62, 63] on the positive relationship between an increase or a reduction in within-day GV and clinical factors, including age, HbA1c, or antidiabetic drugs by 72-h continuous glucose monitoring. However, the two studies did not focus on the stability of within-day GV across the same period. Conversely, we assessed both the overall glycemic control and the within-day GV stability in 499 hospitalized patients with T2D by using 10-day rtCGM data to acquire a more precise evaluation. Additionally, we used %CV_w_([mean SD of all of the measurements in a 24-h period] / [[mean glucose] × 100) to evaluate within-day GV given the existence of three different calculation methods of %CV(%CV_T_, %CV_w_, %CV_b_) for the evaluation of GV. All studies with an evaluation of GV through %CV should specify which %CV was used, according to the study of Julla et al. [23]. However, the calculation method of %CV was not reported in the two studies, although they both evaluated within-day GV through %CV. Furthermore, in our cohorts, the %CV threshold for patients with high within-day GV to be considered as low within-day GV post-treatment was 24%. This was lower than the %CV value (< 27%) reported by Uemura et al. [64] and Monnier et al. [26] to help maintain a minimal risk of hypoglycemia in patients with T2D treated with either insulin and/or noninsulin glucose-lowering agents.

From a clinical standpoint, the findings obtained suggest that most inpatients with T2D struggle to maintain stable, low within-day GV levels, even when they exhibit reasonably good in-hospital glycemic control. Healthcare professionals should recognize potential factors affecting GV that disrupt the balance between glycemic control and glycemic stability, potentially increasing the risk of hypoglycemia. The significance of GV is not widely acknowledged in China currently due to a dearth of relevant recommendations in authoritative guidelines [65]. A prospective observational study drawing from EMR data noted that 71.4% of patients in China were administered insulin upon admission [66]. Nevertheless, our data reveals a strong correlation between insulin therapy and unstable high within-day GV fluctuations; thus, healthcare professionals in China should exercise caution when intensifying glycemic control too aggressively in hospitalized T2D patients. The goal should be to minimize the occurrences of pronounced within-day GV and subsequent hypoglycemia. Furthermore, based on our observations, therapeutic agents that curtail post-meal glucose spikes can enhance within-day GV based on our finding that increased postprandial glucose decreased glycemic stability.

We report several significant limitations in our study. Unlike research grounded in expansive, prospectively gathered datasets, our single-center retrospective data might have variances in accuracy. We were also hamstrung by the absence of specific data on crucial clinical variables, such as the duration of diabetes, due to incomplete records or significant data gaps in the inpatient logs. We should have devoted more scrutiny to the effect of lifestyle factors, like diet and physical activity, and their influence on within-day GV, as such details were absent from our datasets. Moreover, the diverse methods employed to quantify GV can lead to varied conclusions. However, %CV is highly recommended for within-day GV assessments [11].

To conclude, the results highlight the instability of glycemic fluctuations in patients hospitalized with T2D patients and shed light on the existing disparity between glycemic stability and glycemic control in clinical settings. While our findings underscore the imperative for healthcare professionals to prioritize within-day GV in future practices, they also necessitate further investigations conducted within a structured data collection framework to mitigate measurement biases. Our observations also advocate for a measured approach to understanding the interplay between anti-glycemic medications, post-meal glucose surges, and within-day GV instability. This emphasizes the importance of devising improved glycemic management tactics. Further studies are essential to explore therapeutic measures that can effectively reduce post-meal glucose spikes and positively influence within-day GV.

### Electronic supplementary material

Below is the link to the electronic supplementary material.

Supplementary Material 1

## Data Availability

The datasets analysed during the current study are not publicly available due to the hospital’s request and China’s legal regulations on clinical medical data but are available from the corresponding author on reasonable request.

## Abbreviations

HbA1c: Glycated hemoglobin
T2D: Type 2 diabetes
GV: Glycemic variability
rtCGM: Real-time continuous glucose monitoring
BMI: Body mass index
LDL: Low density lipoprotein
FPG: Fasting plasma glucose
PPGE: Postprandial glucose excursion
%CV: Coefficient of variation
SD: Standard deviation
CD: Continuous days
CD after first reaching the target: The consecutive days of maintaining within the target %CV range after first reaching the target
MAX-CD: The maximum consecutive days of maintaining within the target %CV range
DPP-4i: Dipeptidyl peptidase-4 inhibitors
SGLT2i: SGLT2 inhibitors
TZDs: Thiazolidinediones
GLP-1 agonist: Glucagon-like peptide-1 agonist
IQR: Interquartile range
HR: Hazard ratio
IRR: Incidence rate ratios
OR: Odds ratios
CI: Confidence interval
