# Supplementary material for: Assessing the temporal within-day glycemic variability during hospitalization in patients with type 2 diabetes patients using continuous glucose monitoring: a retrospective observational study
Source: Diabetol Metab Syndr. 2024 Mar 1;16:56. doi: 10.1186/s13098-024-01269-0 (PMC10908144; doi:10.1186/s13098-024-01269-0)
Supplement: Supplementary file 1 — Supplementary Material 1 [file 13098_2024_1269_MOESM1_ESM.docx]

| Table S1 Analysis of frequency of within-day GV. | | | | | | | | | | | |
| --- | --- | --- | --- | --- | --- | --- | --- | --- | --- | --- | --- |
| Days during patient's hospital stay | 1st day | 2nd day | 3rd day | 4th day | 5th day | 6th day | 7th day | 8th day | 9th day | 10th day | Total |
| CD after first reaching target^$^ (days) | The number of individuals who had CD after first reaching target^$^ during their hospital stay | | | | | | | | | |  |
| 0 |  |  |  |  |  |  |  |  |  | 55 | 55 |
| 1 |  | 173 | 82 | 65 | 47 | 34 | 19 | 16 | 18 | 14 | 468 |
| 2 |  | 59 | 51 | 29 | 10 | 12 | 10 | 5 |  |  | 176 |
| 3 |  | 33 | 21 | 10 | 5 | 2 | 5 |  |  |  | 76 |
| 4 |  | 15 | 12 | 5 | 4 | 12 |  |  |  |  | 48 |
| 5 |  | 13 | 10 | 6 | 8 |  |  |  |  |  | 37 |
| 6 |  | 13 | 4 | 20 |  |  |  |  |  |  | 37 |
| 7 |  | 6 | 51 |  |  |  |  |  |  |  | 57 |
| 8 |  | 96 |  |  |  |  |  |  |  |  | 96 |
| MAX-CD^#^ (days) | The number of individuals who had MAX-CD^#^ during their hospital stay | | | | | | | | | |  |
| 0 |  |  |  |  |  |  |  |  |  | 55 | 55 |
| 1 |  | 44 | 25 | 34 | 23 | 24 | 17 | 16 | 13 | 14 | 210 |
| 2 |  | 24 | 27 | 29 | 21 | 29 | 33 | 18 | 30 |  | 211 |
| 3 |  | 16 | 12 | 17 | 16 | 16 | 17 | 38 |  |  | 132 |
| 4 |  | 15 | 12 | 9 | 10 | 17 | 61 |  |  |  | 124 |
| 5 |  | 13 | 10 | 8 | 10 | 41 |  |  |  |  | 82 |
| 6 |  | 13 | 4 | 5 | 31 |  |  |  |  |  | 53 |
| 7 |  | 6 | 6 | 30 |  |  |  |  |  |  | 42 |
| 8 |  | 6 | 45 |  |  |  |  |  |  |  | 51 |
| 9 |  | 90 |  |  |  |  |  |  |  |  | 90 |
|  | | | | | | | | | | | |
| The remaining hospitalization days^&^ (days) | 1 | 2 | 3 | 4 | 5 | 6 | 7 | 8 | 9 | 10 |  |
| MAX-CD^#^ (days) | The number of individuals who had MAX-CD^#^ during the remaining hospitalization days | | | | | | | | | |  |
| 0 |  |  |  |  |  |  |  |  |  | 55* | 55 |
| 1 | 14 | 13* | 16* | 17* | 24* | 23* | 34* | 25* | 44* |  | 210 |
| 2 |  | 5 | 4 | 12* | 18* | 22* | 32* | 46* | 72* |  | 211 |
| 3 |  |  | 1 | 2 | 6 | 14* | 24* | 36* | 49* |  | 132 |
| 4 |  |  |  | 3 | 4 | 7 | 15 | 39* | 56* |  | 124 |
| 5 |  |  |  |  | 8 | 3 | 10 | 23 | 38 |  | 82 |
| 6 |  |  |  |  |  | 5 | 4 | 11 | 33 |  | 53 |
| 7 |  |  |  |  |  |  | 16 | 6 | 20 |  | 42 |
| 8 |  |  |  |  |  |  |  | 45 | 6 |  | 51 |
| 9 |  |  |  |  |  |  |  |  | 90 |  | 90 |
| ^$^CD after first reaching target: the consecutive days of maintaining within the target %CV range after first reaching target.  ^#^Max-CD: the maximum consecutive days of maintaining within the target %CV range  *The number of individuals with a proportion of MAX-CD equal to or less than 50%. The proportion of MAX-CD was defined as MAX-CD divided by the remaining days of hospitalization. ^&^The remaining hospitalization days were calculated as the total number of observational days (10 days) minus the number of days required to reach the target %CV range for the first time. | | | | | | | | | | | |


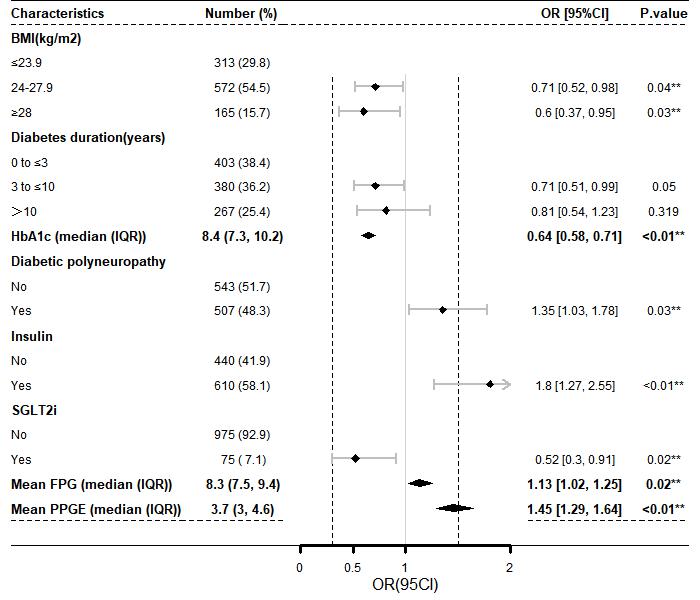


Figure S1. Factors affecting the SD of %CV values. ***P* < 0.05. CI, confidence interval; OR, odd ritio.


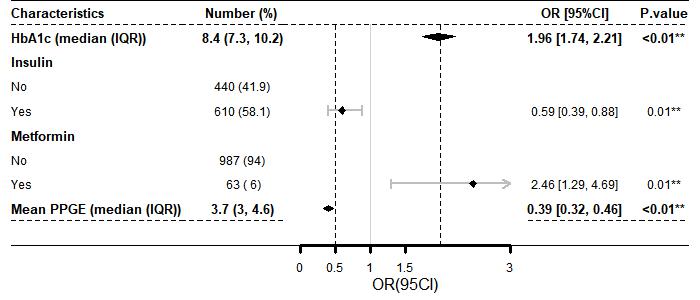


FigureS 2. Factors affecting the proportions of MAX-CD. ***P* < 0.05. CI, confidence interval; OR, odd ritio.
